# MSC Based Therapies—New Perspectives for the Injured Lung

**DOI:** 10.3390/jcm9030682

**Published:** 2020-03-03

**Authors:** Judith Behnke, Sarah Kremer, Tayyab Shahzad, Cho-Ming Chao, Eva Böttcher-Friebertshäuser, Rory E. Morty, Saverio Bellusci, Harald Ehrhardt

**Affiliations:** 1 Department of General Pediatrics and Neonatology, Justus-Liebig-University, Universities of Giessen and Marburg Lung Center (UGMLC), German Center for Lung Research (DZL), Feulgenstrasse 12, 35392 Gießen, Germany; Judith.Behnke@paediat.med.uni-giessen.de (J.B.); Sarah.Kremer@paediat.med.uni-giessen.de (S.K.); Tayyab.Shahzad@paediat.med.uni-giessen.de (T.S.); Cho-Ming.Chao@paediat.med.uni-giessen.de (C.-M.C.); 2 Department of Internal Medicine II, Universities of Giessen and Marburg Lung Center (UGMLC), Cardiopulmonary Institute (CPI), German Center for Lung Research (DZL), Aulweg 130, 35392 Giessen, Germany; Saverio.Bellusci@innere.med.uni-giessen.de; 3 Institute of Virology, Philipps University Marburg, Hans-Meerwein-Strasse 2, 35043 Marburg, Germany; friebertshaeuser@staff.uni-marburg.de; 4 Department of Lung Development and Remodeling, Max Planck Institute for Heart and Lung Research, German Center for Lung Research (DZL), Ludwigstrasse 43, 61231 Bad Nauheim, Germany; rory.morty@mpi-bn.mpg.de

**Keywords:** mesenchymal stem cells, MSC, chronic lung disease, lung repair, extracellular vesicles, bronchopulmonary dysplasia, asthma, chronic obstructive pulmonary disease, idiopathic pulmonary fibrosis, inflammation, lung injury

## Abstract

Chronic lung diseases pose a tremendous global burden. At least one in four people suffer from severe pulmonary sequelae over the course of a lifetime. Despite substantial improvements in therapeutic interventions, persistent alleviation of clinical symptoms cannot be offered to most patients affected to date. Despite broad discrepancies in origins and pathomechanisms, the important disease entities all have in common the pulmonary inflammatory response which is central to lung injury and structural abnormalities. Mesenchymal stem cells (MSC) attract particular attention due to their broadly acting anti-inflammatory and regenerative properties. Plenty of preclinical studies provided congruent and convincing evidence that MSC have the therapeutic potential to alleviate lung injuries across ages. These include the disease entities bronchopulmonary dysplasia, asthma and the different forms of acute lung injury and chronic pulmonary diseases in adulthood. While clinical trials are so far restricted to pioneering trials on safety and feasibility, preclinical results point out possibilities to boost the therapeutic efficacy of MSC application and to take advantage of the MSC secretome. The presented review summarizes the most recent advances and highlights joint mechanisms of MSC action across disease entities which provide the basis to timely tackle this global disease burden.

## 1. Introduction

Together with cardiovascular diseases and cancer, lung diseases across all ages are one of the three leading causes of acute and chronic morbidity and mortality in the world. More than 500 million people from low- and middle-income countries suffer from chronic respiratory diseases with tremendous negative impact on health status, economic situation of the family, socioeconomic costs and quality of life. For chronic obstructive pulmonary disease (COPD) alone, total annual costs in the United States (in 2000) are estimated to outreach $32 billion per year [1]. Despite all preventive measures, incidences are increasing steadily, and children and older people are particularly affected. Moreover, chronic lung diseases account for more than four million annual deaths worldwide. Only 20% of cases with chronic diseases have regular access to specialized care services within the industrialized world [2]. Despite the tremendous progress in mechanistic understanding of disease pathogenesis, for most of them no curative approach is available. So far, therapeutic interventions have the goal to alleviate clinical symptoms and to delay disease progression. During the recent years, cell-based therapies have attracted particular attention. Research results triggered an unforeseen enthusiasm about this new approach [3]. This review summarizes the major results of mesenchymal stem cell (MSC) based therapies to the lung. It points out common mechanisms of MSC action across disease entities which represent particularly well-suited approaches to tackle this tremendous global disease challenge. It is dedicated to highlighting the important advances during the last five years. Furthermore, it gives a perspective on future research directions based on the latest research advances which have the power to accelerate therapy access and to improve therapeutic safety and efficiency.

## 2. The Therapeutic Potential of MSC

Not only during development but throughout the whole life span, stem cells are critical for physiologic maintenance and organ repair in case of injury. This has also been studied extensively for the lung. Phenotype distortion and rarefication of pulmonary stem cells represent key features of any lung pathology and central drivers to lung injury in amongst others including bronchopulmonary dysplasia (BPD), acute lung injury (ALI) and COPD, as well as towards aging. Therefore, the idea of restoration of physiologic cell function fascinates researchers around the world [4,5,6,7,8]. Organ repair is not restricted to resident stem cells. Stem cells from the bone marrow and other tissues can be attracted to the site of injury to contribute to organ repair and have the capability to foster endogenous progenitor cell function in the lung [9].

For this reason, the therapeutic potential of MSC to recover the injured lung has been the subject of extensive research efforts during the last decade [3]. MSC possess multipotent and reparative functions and can be accessed from nearly every postnatal tissue. Their easier accessibility, improved safety profile and nonexistent ethical concerns makes them a superior candidate for stem cell therapy compared to embryonic stem cells and induced pluripotent stem cells [3]. Special attractiveness arises from the immunoprivileged status of MSC. They do not trigger a host response or cell rejection response because they are less sensitive to pro-inflammatory IFN-γ-induced HLA-II expression [10].

**Figure 1:**
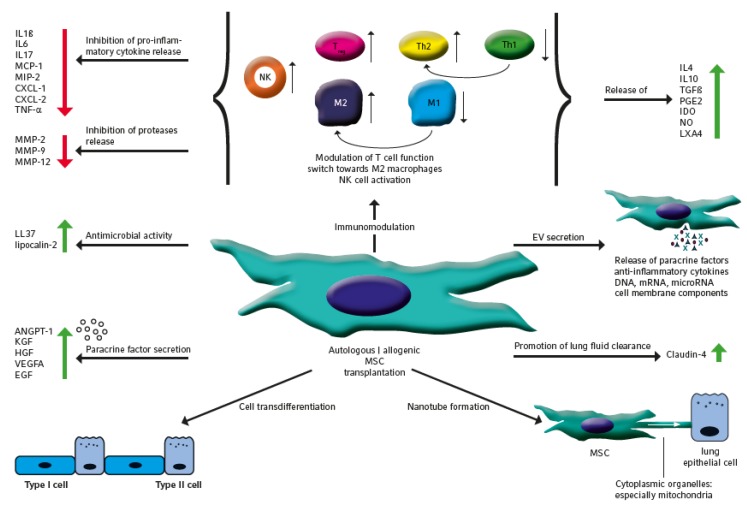
Multiple lung repair and regenerative functions of mesenchymal stem cells (MSC). Autologous or allogenic MSC from different rodent species and humans proved therapeutic efficacy in different preclinical lung disease models mostly studied in rodents. The graphic summarizes the multiple actions of MSC transplantation to the injured lung including cell transdifferentiation, secretion of paracrine factors, antimicrobial activity, immunomodulation, EV secretion, promotion of lung fluid clearance, nanotube formation and cell component transfer. MSC immunomodulation comprises T-cell, macrophage and NK cell function resulting in the augmented release of anti-inflammatory cytokines and beneficial factors, such as the inhibition of proteases as well as attenuation of pro-inflammatory cytokine releases. IL—interleukin; MCP-1—monocyte chemoattractant protein-1; MIP-2—macrophage inflammatory protein-2; CXCL—C–X–C motif ligand; TNF-α—tumor necrosis factor alpha; MMP—matrix metallopeptidase; ANGPT-1—angiopoietin 1; KGF—keratinocyte growth factor; HGF—hepatocyte growth factor; VEGFA—Vascular endothelial growth factor A; EGF—epidermal growth factor; NK—natural killer cell; Treg—regulatory t cell; Th—t helper cell; MSC—mesenchymal stem cell; TGFß—transforming growth factor beta; PGE2—prostaglandin E2; IDO—indoleamine-pyrrole 2,3-dioxygenase NO—nitric oxide; LXA4—lipoxin A4; EV—extracellular vesicle.

To date, studies on MSC therapies involved cells from the bone marrow, peripheral blood, umbilical cord, Wharton’s jelly, placenta and adipose tissue and cells from newborns and adults [11]. MSC comprise plenty of lung regenerative and repair functions that are highlighted within the subsequent sections and are summarized in Figure 1.

Initially, MSC action was attributed to cell transdifferentiation and replacement of resident cells at the site of injury. The beneficial effects of cell differentiation were proven e.g., in ALI, but overexpression of certain microRNAs (e.g., microRNA-615-3p or microRNA-155-5p) in the recipient lung prevents the differentiation of MSC into type II cells attenuating therapy efficacy and leading to disease progression [12,13].

Due to the overwhelming data on MSC paracrine functions, this concept as primary action of MSC has been omitted and research directions were focused towards the paracrine effects of MSC. Paracrine efficacy was supplemented by the observation that MSC only sparsely and temporarily engraft in the injured lung, although better MSC engraftment was observed in the injured lung and engraftment rates increased with the extent of tissue injury [14]. Moreover, MSC secrete plenty of growth promoting and regeneration stimulating factors amongst others, such as angiopoetin 1 (ANGPT1), keratinocyte growth factor (KGF), hepatocyte growth factor (HGF), epidermal growth factor (EGF) and vascular endothelial growth factor alpha (VEGFA), which were identified to centrally drive repair and regeneration. On a molecular level, KGF and HGF secretion directly protect alveolar epithelial cells from apoptosis under hypoxic conditions by stabilization of endogenous Bcl-2, inhibition of HIF1α protein expression and reactive oxygen species (ROS) production [15]. Further identified factors comprise a heterogeneous group including Lipoxin A4 and the epithelial protection factor secretory leukocyte protease inhibitor (SLPI) reduced elastin breakdown by proteases [16,17]. Besides the release of lung growth stimulating factors, the paracrine activity of MSC was mainly traced back to the inhibition of immune cell attraction to the site of injury and to their immunomodulatory properties by both inhibiting the innate and adaptive immune response. In addition, the functionality of exogenous MSC on immunomodulation of lung diseases is much better understood than the interaction of lung resident MSC with the immune system. MSC exert direct interaction with immune cells and execute paracrine modulation of the immune response by the release of cytokines like TGF-β, IL-10 and IL-1RA and the production of nitric oxide and indoleamine 2,3 dioxygenase (IDO) [18]. These two main mechanisms modulate the proliferation and activation of naïve and effector T cells, NK cells and mononuclear cells towards an anti-inflammatory phenotype. Modulation of T cell function includes the inhibition of the Th17 response, the induction of CD4+CD25+FoxP3+ Treg cells and the shift from a Th1 to the Th2 cell phenotype. This process is regulated context-specifically by anti-inflammatory cytokines including IL-10 and TGF-β, growth factors such as HGF, further soluble factors like PGE2 and the inhibition of disease-driving cytokines like IL-4 and IL-13 [19,20]. Likewise, MSC shift the phenotype and function of antigen-presenting cells and includes dendritic cells, B lymphocytes and macrophages and prevention of neutrophil extracellular trap formation [21]. The phenotype change in macrophages leads to a switch back from the M1 state to the “anti-inflammatory” M2 status. This switch is induced by the inflammatory milieu and bacterial infection and fosters inflammation by the release of a plenty of pro-inflammatory factors including cytokines and proteolytic enzymes. Reversely, the M2 macrophages have important functions in limiting inflammation and myeloperoxidase activity, and in phagocytosis and tissue repair. The long-lasting induction of immune tolerance in lung macrophages seems to occur independent of Treg function but is ascribed towards induction of adaptive innate tolerance mediated by TNF-stimulated gene 6 protein [19,21]. As the result of immunomodulation, MSC shift inflammation from an overwhelming release of pro-inflammatory cytokines including IL-1β, IL-6, MCP-1, MIP-2, CXCL-1, CXCL-2, TNF-α, IL-12, IL-17 or type II IFN-γ and proteases like MMP-2, MMP-9 and MMP-12 to an anti-inflammatory status with overweight of IL-4, IL-10, TGF-β, CCL18, prostaglandin E2, IDO, nitric oxide and inflammation resolving lipoxin A4 (LXA4), which enable the resolution of inflammation and endogenous tissue repair [22,23].

MSC display further disease limiting capacities. These include antimicrobial activity, inhibition of epithelial-mesenchymal transition and lung fluid clearance; during infection, MSC secret antimicrobial factors like peptide LL-37 and lipocalin-2, stimulate the phagocytosis activity of macrophages in the lung and improve the clearance of bacteria [24,25,26]. MSC possess the capability to prevent epithelial-mesenchymal transition of alveolar epithelial cells which is regulated by inhibition of TGF-β1 gene transcripts including ZEB1, TWIST1 and CTGF and is partly mediated by KGF [27]. Promotion of alveolar fluid clearance by MSC depends on claudin-4 which belongs to a family of proteins centrally involved in tight junction formation [28].

Impaired energy supply is a further key feature of acute lung injury. Formation of gap junctional channels and direct transfer of cytoplasmic organelles via connexin 43, especially mitochondria, stabilizes cell energetics and has been identified as another mechanism of MSC action. This transfer increases alveolar epithelial ATP and attenuates vascular permeability and influx of inflammatory cells [29]. In this context Miro 1 was identified as the crucial nanotube connector between lung epithelial cells and MSC [30]. Underlining the importance, transfer of extracellular vesicle (EV) defective mitochondria was unable to alleviate lung injury and to improve survival. Cell-cell contact and transfer of cellular material can take place to structural lung cells or immune cells and intercellular transfer to macrophages prevented the accumulation of inflammatory macrophages and the production of inflammatory and pro-fibrotic cytokines like TNF-α and TGF-β [31].

Last but not least, MSC action has been ascribed to intercellular communication via extracellular vesicle release, which comprises the so-called exosomes and microvesicles which can be differentiated by size, the mechanisms of excretion and the content of cellular components [32]. This mechanism comprises the exchange of proteins including growth factors and anti-inflammatory cytokines, lipids, DNA, mRNA, microRNA and cell membrane components including surface receptors and molecules and thereby shapes the phenotype and functional properties of recipient cells. Several cytokines and growth factors including KGF, ANGPT1, EGF, HGF and SDF-1 have been identified as main drivers of EV action. To confirm this central role, transfection experiments with siRNA against KGF were carried out before vesicle harvest which resulted in reduced anti-inflammatory and lung protective activity during acute lung injury [33]. Due to its special therapeutic importance, the function and therapeutic application of EV is described in detail within the second last paragraph of this review.

Within recent years, there has been tremendous progress in understanding the similarities and inhomogeneities of MSC from different tissues and the important role of aging on oxidative stress and DNA damage response. Besides MSC from the bone marrow, the research focus was directed towards “young” MSC from umbilical cord blood and the Wharton’s Jelly which possess superior anti-inflammatory and immunomodulatory properties [11]. Due to the limitations in space, these discrepancies will not be presented in detail. They will be tackled within the specific disease area sections where concise data were retrieved during the recent years.

## 3. Therapeutic Efficacy of MSC Application in Preclinical Lung Disease Models

### 3.1. Bronchopulmonary Dysplasia

Bronchopulmonary dysplasia (BPD) is characterized by distortion of further alveolar, mesenchymal and vascular lung development in the saccular stage. This developmental derangement is caused by the deleterious insults of infection, mechanical ventilation and oxygen toxicity which induce a pulmonary inflammatory response in the immature lung [5]. In the preclinical model of BPD with exposure of rodent pups to hyperoxia, MSC was effective in reverting lung injury by attenuating the influx of inflammatory cells to the lung, pro-inflammatory cytokine accumulation and by improving alveolar and vascular lung structure and function [34,35]. Comparable reversal of pathologic features is described for the intratracheal and systemic route of MSC application [34,35]. Even retarded MSC application two weeks after injury prevailed in reverting tissue distortion which underlines the central contribution of paracrine cytokine and growth factor secretion to lung repair and regeneration [36]. Of interest, MSC from female donors displayed a stronger effect on vascular remodeling [37]. Within the detailed investigations of disease-driving pathologies, macrophage shifts towards an anti-inflammatory phenotype were identified as one of the key features following MSC application [38]. The available studies on rodent BPD models were recently summarized in a meta-analysis and the authors came to the conclusion that MSC impacted on all relevant outcome parameters including lung inflammation, fibrosis and apoptosis, alveolarization, angiogenesis and pulmonary hypertension. Therapeutic efficacy was similar for the preventive and the rescue application and improvements in lung structure persisted until adulthood [39,40]. Neither the timing, the source or amount of MSC applied nor the route of administration determined the effect size [39]. Of special notice, effects of intratracheal application were not restricted to the lung but led to a reduction of brain injury. As for the lung, the main action in the brain was attributed to attenuation of inflammation and apoptosis induction [41]. A compilation of pioneering studies is provided in Table 1 which presents all details on model species, MSC source, route and timing of MSC application [42,43].

### 3.2. Asthma

Asthma is a chronic inflammatory disorder of the airways provoked by endogenous (e.g., chronic inflammation and oxidative stress) and exogenous factors (e.g., exposure to cigarette smoke and air pollution) leading to airway hyperresponsiveness, airflow limitations and finally persistent changes in airway structure. In contrast to most other lung diseases, different disease subtypes are well acknowledged that can be separated by different inflammatory disease patterns. The first descriptions of MSC therapy in allergic asthma models revealed attenuation of all areas of pathology including airway hyperresponsiveness, mucus production, immunologic response, inflammatory cell infiltration, lung function, remodeling and serum IgE levels [44,45]. Central actions of MSC are the reduction of eosinophilia and neutrophil infiltration, a Th1/Th2 shift with modulation of the adaptive arm of Th2 immunity, the inhibition of TH2 cytokine release including IL-4, IL-5 and IL-13 and Treg cell induction. MSC action is not restricted to T cell function but as well restricts the number of activated dendritic cells [45,46,47,48,49,50,51]. Likewise, MSC suppress dendritic cell function by arresting them in an immature state, unable to be used for antigen presentation and T cell activation [49]. MSC fundamentally alter airway inflammatory cell pathology with the recruitment of CCR2+ monocytes but reduce attraction of neutrophils thereby opening a therapeutic option also for the steroid-resistant asthma phenotype [52,53]. The M2 macrophages centrally account for the MSC effects in experimental asthma due to their anti-inflammatory properties, immunosuppressive functions with high levels of IL-10 and TGF-β and high phagocytosis capacity, especially in situations where T cells are already activated and thereby prohibit Treg cell expansion [54,55]. The conflicting data on Treg cell activation and functionality by MSC might be explained by the different experimental settings and the different sources of MSC from rodents and humans applied. Downstream action of MSC on inflammation and airway remodeling was traced back to inhibition of PI3 kinase/AKT and modulation of Notch signaling [56,57].

Recent investigations showed better or exclusive therapeutic efficiency by bone marrow-derived (BM) MSC but not adipose tissue-derived MSC in the ovalbumin model where structural and functional outcome parameters were redirected towards the physiologic situation only by bone marrow MSC [58]. Combined application together with simvastatin with the aim to foster the migration of MSC to the site of inflammation and lung injury proved superior compared to each treatment alone [50].

Taken together, MSC therapy in experimental asthma addresses all disease-relevant pathologies which are compiled for selected pioneering studies in Table 2.

### 3.3. Acute Lung Injury

Acute lung injury is caused by plenty of different acute insults to the lung including bacterial and viral infections, inhalation of toxic agents but also systemic causes leading to an acute and mostly dramatic deterioration or failure of gas exchange [59]. Local or systemic MSC treatment markedly reduced alveolar permeability and lung inflammation in acute lung injury induced by Lipopolysaccharides (LPS). The results were reproduced for a human lung perfusion model [60,61]. MSC therapy following acute lung injury attenuated inflammation and lung tissue remodeling and functional relevance was confirmed by lung function testing [62,63]. ANGPT1 was identified as one central factor which promoted endothelial cell survival and reduced vascular permeability. In addition, KGF was determined as the second driver of MSC action [64,65]. Furthermore, Lipoxin A4 plays a central role in the resolution of inflammation by inhibition of TNF-α and MIP-2 production. Vice versa, inhibition of Lipoxin A4 prohibits its anti-inflammatory effects, offsetting the increased survival in ALI [16]. While TLR3 is counterproductive, the activation of TLR4 on MSC enhances lipocalin-2 activity, suppresses bacterial growth and promotes bacterial clearance [66]. Described so far only for ALI, neutrophil extracellular trap formation was prohibited by MSC [21]. In addition, beneficial effects of MSC were attributed to bacterial growth inhibition, promotion of phagocytosis of gram-positive and gram-negative bacterial strains and increased expression of the alveolar cathelicidin antimicrobial peptide LL-37, a host defense peptide with a wide range of immunomodulatory activities and modest direct antimicrobial properties [24,25,67].

EV-mediated mitochondrial transfer to alveolar epithelial cells was first identified to ameliorate acute lung injury. CD44 expressing EV mediated mitochondrial transfer and promoted an anti-inflammatory and phagocytic phenotype of macrophages which was centrally attributed to the amelioration of lung injury [29,68,69]. Blockade of mitochondrial nanotube transfer abrogated improved macrophage bioenergetics and MSC effects including promotion of phagocytosis capacity [68], but MSC action displays some specific differences between different pathogens. For pseudomonas aeruginosa infection, the beneficial effects were specifically ascribed towards a systemic IGF-1 mediated inhibition of COX-2 and prostaglandin E2 production and promotion of 15-hydroxyprostaglandin dehydrogenase activity while lung lavage levels of known beneficial factors like KGF, ANGPT-1 and IGF-1 were not changed [23]. In ALI after *E. coli* ingestion, the beneficial effects of MSC were specifically assigned to the population of CD362+ MSC [70]. Despite these specificities, broadly comparable beneficial effects on inflammatory cytokine response, influx of inflammatory cells, lung histopathology and survival were evident in mice and rats and for different MSC cell preparations from mice and men. Routes of administration or time points of therapeutic intervention before and after LPS injury did not relevantly impact therapeutic efficacy [33,60,67,71,72]. The secretome of cryopreserved human MSC was less effective in an *E. coli* rat model, underlining the need for application of freshly isolated cells for maximum benefit at least in some ALI situations. Then, cell dosages as low as 5x10^5^ cells per kilogram body weight were effective [66].

Besides bacterial pneumonia, MSC application proved therapeutic efficiency during influenza infection resulting in reduced impairment of alveolar fluid clearance and lung injury. This was attributed towards attenuation of pro-inflammatory cytokine secretion, inflammatory cell recruitment and increased alveolar macrophages content [73,74]. Again, ANGPT-1 and HGF were identified as key mediators of MSC action. Paracrine factor release was more efficient for MSC from the umbilical cord than from the bone marrow [75]. The beneficial effects were detectable for simultaneous or one day delayed MSC application. The universal beneficial effects for different influenza strains are not in accordance with the underlying pathomechanisms as ANGPT-1 and KGF regulation was restricted to the H5N1 species [73]. These discrepancies might be deducted to the highly inflammatory phenotype provoked by H5N1 but not H1N1, making H5N1 a better candidate for MSC based interventions [76].

A summary of pioneering studies on different ALI models is compiled in Table 3 which includes details on differences between disease models, species, MSC source, route and timing of MSC application [77].

### 3.4. Chronic Obstructive Pulmonary Disease

COPD constitutes one of the most frequent chronic lung diseases of mankind with manifold morbidities and high mortality. Pathogenesis is dominated by airway inflammation and lung fibrosis leading to airway obstruction and emphysema. The multifactorial origin including genetic and gender predisposition is dominated by smoke exposure [78]. Nowadays, immunosuppression by corticosteroids is standard of therapy and attenuates disease severity. The dual anti-inflammatory and regenerative action of MSC together with their ability of attraction to the site of lung injury makes them a highly promising future candidate for efficient therapy of COPD. First results from the smoke model demonstrated impressive reversal of lung architecture destruction which stipulated a large number of subsequent preclinical trials [79,80,81]. MSC therapy in COPD models of both elastase or cigarette smoke exposure in mice and rats reduced tissue destruction and emphysematic changes. This was mediated by decreasing the inflammatory response with attenuated M1 macrophage cytokine release including IL-1β, IL-6, TNF-α and MCP-1 and protease activity responsible for elastic fiber degradation and tissue remodeling. The parallel increase in HGF, EGF, SLPI, VEGFA, nuclear factor erythroid 2-related factor (Nrf 2) and TGF-β activity and superoxide dismutase led to decreased tissue changes and lung cell apoptosis [56,82,83]. Endothelial cell dysfunction constitutes a key feature of COPD. A specific preventive function has been described for VEGFA [84]. Recent findings hint towards a central role of cyclooxygenase-2 mediating prostaglandin E2 production by p38 and ERK MAP kinase signaling and airway inflammation in alveolar macrophages which shift the balance towards an M2 state [85]. Besides transdifferentiation into type II cells, the prevention of AT II cell apoptosis was identified as another key feature of action which was attributed to the overexpression of antiapoptotic proteins and suppression of caspase activity [14,85,86]. Furthermore, mitochondrial transfer via nanotube formation seems to constitute another mode of MSC action which is hampered by injury due to cigarette smoke exposure [87].

As for BPD and ALI, different origins of MSC cell preparations, variations in the timing and dosing of MSC, route of application and studies in various rodent models resulted in congruent results of mechanistic action with primary impact on inflammation and airway enlargement. Therapeutic efficacy and systemic effects varied between different cell preparations and routes of administration [17,83,85,88]. Despite the observed congruencies, disparities were observed for the important outcome measures of macrophage polarization and cardiovascular function with superiority of bone marrow-derived MSC compared to adipose tissue MSC [88]. MSC were only effective during the acute stage of lung inflammation. In the chronic situation of established emphysema, no beneficial effects of MSC application were recorded [89]. Repeated application proved superiority with respect to immunologic cell pathology in the lung and pulmonary arterial hypertension [90]. For overview purposes pivotal studies on different COPD models are assorted in Table 4 [91,92,93].

### 3.5. Idiopathic Pulmonary Fibrosis

Within the heterogeneous category of interstitial lung diseases, idiopathic pulmonary fibrosis (IPF) represents the most frequent disease which is characterized by excess fibrosis and remodeling. Its low incidence qualifies it as a rare disease, but the global disease burden is immense [6,94]. Initiation and progression of disease pathogenesis are characterized by inflammation and fibroblast dysfunction which results in lung fibrosis. In contrast to other lung diseases, M2 macrophages, TH17 and Th2 cells play a dominant role during disease evolution. The Th2 cells centrally release cytokines such as IL-4, IL5 and IL-13. IL-13 constitutes the central driver of myofibroblast activation and tissue remodeling. Further complexity arises from the fact that immune cells like Treg cells provide harm if they preferably secrete TGF-β but exert a protective function if IL-10 is primarily secreted [94]. Moreover, CD4+ T cells were identified as another central target of MSC action leading to attenuated inflammation and lung fibrosis [95]. In the preclinical rodent model, MSC application in the acute phase of disease suppressed proinflammatory cytokine production, the release of nitric oxide and TGF-β from immune cells and resident alveolar macrophages and suppressed the production of profibrotic and inflammatory genes [96,97,98]. In this experimental context, the interleukin 1 receptor antagonist expressing MSC subpopulation was identified to exert all beneficial effects observed and specifies the MSC fraction of action [99]. Besides abrogation of TGF-β signaling, inhibition Wnt/β-catenin signaling and thereby inhibition of myofibroblast differentiation of lung resident MSC and inhibition of pro-fibrotic mir-199-3p upregulation were identified as further central mechanisms of action [100,101]. The therapeutic efficiency depends on the age of MSC applied and old donor cells were not able to impact on key regulations like MMP-2, IGF receptor and AKT activation [102]. However, MSC application proved more efficient than the actual clinical standard therapy with pirfenidone [98]. Early application of MSC during the initiation of lung inflammation is the decisive factor as this approach has the potential not only to alleviate lung inflammation but also lung fibrosis, while retarded application after disease establishment was only capable to attenuate inflammation but failed to reduce lung fibrosis [103]. The important insights on MSC action in IPF are detailed in Table 5 and display the congruencies and disparities to other pulmonary diseases [104].

MSC therapy has been extended to a number of further lung diseases including radiation-induced lung injury and ischemia reperfusion damage during lung transplantation. Due to the limitations in space these diseases were not included in the detailed presentation but key beneficial effects of MSC therapy have been summarized elsewhere [94,105,106].

Due to the still limited therapeutic options in lung diseases, the high therapeutic efficiency of MSC in preclinical lung disease models across all ages to attenuate inflammation and fibrosis raises great enthusiasm. Comparability of results is still hampered by the broad heterogeneity of published results with respect to preventive or rescue MSC therapy, the cell type, dosage and route of MSC application as displayed in Table 1, Table 2, Table 3, Table 4 and Table 5, but congruency of efficacy argues towards a broad range of therapeutic options. Future research efforts need to be directed towards the optimal therapy conditions and the window of therapy is directed to the acute phase of lung injury. Abnormal alveolar epithelial cell function and immunologic dysregulation with the release of cytokines and proteinases leads to lung injury, abnormal wound repair, lung fibrosis and rarefication, and myofibroblastic differentiation of lung MSC which can all be reverted by exogenous MSC. Most of the studies were performed in rodents and within established animal disease models that usually display a severe phenotype but do not reflect genetic or inter-strain variability or do not mimic the origins of lung disease as, for example, for the bleomycin IPF model [107,108]. Surprising only at the first site, the mechanisms of MSC action are largely homologous. This underlines the potential for unique approaches and the option for common study readouts across disease entities to speed up the dissemination of this promising therapy into clinical trials.

**Table 4:** Summary of important preclinical studies in rodents studying the therapeutic potential of MSC for COPD.

Experimental Lung Disease Model	Species	Cell Source	MSC Species	Dose (Cells)	Application Route	Time Point of Application	Repeated Application	Biological Function In Vivo	Molecular Changes/Results	Reference
cigarette smoke	rat	BM	rat	6 × 10^6^	i.t.	beginning 7th week	yes (2x/week for 5 weeks)	alleviated airway inflammation and edema	downregulated COX2 and prostaglandin E production suggested to be mediated by inhibition of p38 and ERK MAP kinase activity in macrophages	[85]
elastase	mice	BM	mice	1 × 10^5^ vs. 2 × 10^5^	i.t.	3 h p.i. (second application after 7 days)	yes	reduced inflammation and collagen fiber content, improved VEGFA secretion and lung mechanics	only the repeated application one week apart reduced neutrophil counts and T cell pathology resulting in attenuation of pulmonary arterial hypertension	[90]
cigarette smoke	rat	BMC vs. BM	rat	6 × 10^6^ vs. 6 × 10^5^	i.v.	6 month a.t.	no	BMC and MSC increased pulmonary vascularity, cell proliferation and number of small vessels. BMC reduce apoptotic cell death, attenuate mean pulmonary arterial pressure and muscularization	BMC better induced proliferation of AT2 cells and pulmonal vascular endothelial cells, BM MSC and BMC both alleviate emphysema.	[81]
elastase	rat	AT	rat	5 × 10^7^	local (PGAF)	1 week a.t.	no	enhancement of compensatory growth, restoration of pulmonary function, alveolar and vascular regeneration	selective delivery of HGF by AT MSC with alveolar regenerative and angiogenic effects	[79]
papain	rat	BM	rat	4 × 10^6^	i.v.	2 h a.t.	no	protective effect on pulmonary emphysema by secretion of reparative growth factors	BM MSC increase VEGF-A expression by TNF-α release with inhibition of apoptosis	[80]
elastase	mice	WJ	human	5 × 10^4^	i.v.	7 d a.t.	no	reduced degree of alveolar emphysema	WJ MSC deliver pulmonary regenerative effect, pathomechanism not investigated	[91]
elastase	mice	LT	mice	5 × 10^4^	i.t.	21 d a.t.	no	partially restored lung elasticity and alveolar architecture	activation of HGF/c-Met system, by promoting survival and proliferation of alveolar epithelial cells	[92]
cigarette smoke	mice	AT	human vs mice	3 × 10^5^	i.v.	during last 8 weeks of treatment	yes (4x/last 8 weeks of treatment, biweekly)	reduced inflammatory infiltration, decreased cell death and airspace enlargement and restored weight loss	AT MSC abrogated the phosphorylation of p38 MAPK and attenuated JNK1 and AKT1 activities, murine and human ASC have same effects	[93]
elastase	mice	BM vs AT vs LT	mice	1 × 10^5^	i.v. vs. i.t.	3 h a.t.	no	BM, AT and LT MSCs decreased mean linear intercept, neutrophil infiltration, and cell apoptosis, increased elastic fiber content, reduced alveolar epithelial and endothelial cell damage	decreased keratinocyte-derived chemokine and TGF-β levels in all sources, i.v. administration of BM MSC with better cardiovascular function and phenotype change from M1 to M2	[88]
elastase or cigarette smoke	mice	AT	human	1 × 10^5^	i.v.	7 d a.t. vs. 6-month a.t.	no	improved alveolar regeneration	AT MSC decrease mean linear intercept and reduce caspase-3 activity	[109]

i.v.—intravenous; i.t.—intratracheal; a.t.—after treatment; MSC—mesenchymal stem cell; COPD—chronic obstructive pulmonary disease; BM—bone marrow-derived MSC; AT—adipose tissue- derived MSC; BMC—bone marrow-derived mononuclear cells; PGAF—polyglycolic acid felt sheet; WJ—whartons’s jelly-derived MSC; LT—lung tissue-derived MSC; h—hours; d—day; COX2—cyclooxygenase-2; ERK—extracellular signal-regulated kinase; MAP—mitogen-activated protein kinase; HGF—hepatocyte growth factor; VEGF-A—vascular endothelial growth factor A; TNF-α—tumor necrosis factor alpha; ATII—alveolar epithelial type II; JNK1—c-Jun N-terminal kinase 1; AKT1—AKT serine/threonine kinase 1; TGF-ß—transforming growth factor beta.

**Table 5:** Summary of important preclinical studies in rodents on the therapeutic efficacy of MSC to treat Idiopathic pulmonary fibrosis.

Experimental Lung Disease Model	Species	Cell Source	MSC Species	Dose (Cells)	Application Route	Time Point of Application	Repeated Application	Biological Function In Vivo	Molecular Changes/Results	Reference
bleomycin	mice	BM	mice	5 × 10^5^	i.v.	1 d vs. 3 d vs. 6 d a.t.	no	lung fibrosis and inflammation inhibited to greater degree in day 3 and 6, later administration of BM MSC engraftment more effective	decreased MMP9, TIMP-1, IFN-γ and TGF-β activity	[104]
bleomycin	mice	AT	mice	5 × 10^5^	i.v.	1 d a.t.	no	AT MSC from aged mice are inefficient to attenuate disease pathology	AT MSC from young mice inhibit MMP-2, IGF receptor and AKT activation	[102]
bleomycin	mice	AT	human	4 × 10^7^/kg BW	i.v.	3,6,9 d a.t.	yes	increased survival, reduced collagen deposition, immunomodulation and anti-fibrotic effect in early stage of disease	suppression of profibrotic and inflammatory gene transcripts	[98]
bleomycin	mice	BM	human	5 × 10^5^	i.v.	2 d a.t.	no	reduced pulmonary fibrosis and improved lung function	suppression of total T cell and CD4+ T cell infiltration, pro-inflammatory cytokine production and fibrotic changes	[95]
bleomycin	mice	AT	mice	5 × 10^5^	i.v.	1 d a.t.	no	attenuation of lung fibrosis	inhibition of mir-199-3p and AKT phosphorylation, preservation of caveolin-1 expression	[101]
bleomycin	mice	BM	mice	5 × 10^5^	i.v.	7 d a.t.	no	reduction of inflammation and collagen deposition	response to injury, adopt an epithelium-like phenotype, and reduce inflammation and collagen deposition, replacing alveolar epithelial type II cells, reduced expression of MMP2 and MMP9	[96]
bleomycin	rat	BM	rat	1 × 10^6^	i.v.	4 d a.t.	no	reduced lung injury and fibrosis, lower neutrophilic infiltration and collagen deposition	down-regulation of IL-1β, TGF-β VEGF, IL-6, TNF-α, and NOS	[97]
bleomycin	mice	BM	mice	5 × 10^5^	i.v.	n.a.	no	protection of lung injury	Secretion of high levels of IL1RN, to antagonize the function of IL-1α and block production of TNF-α from activated macrophages	[99]

i.v.—intravenous; a.t.—after treatment; MSC—mesenchymal stem cell; ALI—acute lung injury; BPD—bronchopulmonary dysplasia; COPD—chronic obstructive pulmonary disease; BM—bone marrow-derived MSC; AT—adipose tissue-derived MSC; BW—birth weight; d—day; n.a. —not applicable; MMP—matrix metallopeptidases; TIMP-1—tissue inhibitor of metalloproteinase 1; IFN-γ—interferon gamma; TGF-β—transforming growth factor beta; AKT—AKT serine/threonine kinase; IL—interleukin; VEGF—vascular endothelial growth factor; TNF-α—tumor necrosis factor alpha; NOS—nitric oxide synthase; IL1RN—interleukin 1 receptor antagonist.

## 4. Summary of Important Clinical Phase I and Phase II Studies

The unprecedented positive results from the preclinical studies in rodents across all pulmonary disease entities encouraged the introduction of MSC therapy into the clinics within mainly phase I trials. We will focus in this paragraph on these pioneering trials where patient outcome data are already available. Actually, many more larger trials are ongoing that are powered to better estimate the therapeutic potential of MSC for the treatment of lung diseases. They will give more reliable responses to the most appropriate source, optimum dose and route of MSC application [5].

The first study of MSC therapy was performed in *n* = 12 patients for the treatment of acute respiratory distress. It did not prevail any acute toxicity and serious adverse events were not more frequently observed in the intervention group. The unchanged cytokine profile in serum samples was interpreted as missing therapeutic efficiency that was attributed to the low amount of MSC applied [110]. Another study in *n* = 9 patients revealed two deaths, one of them attributed to multiple embolism that were not attributed to MSC therapy [111]. Further phase I and phase II studies are currently ongoing and powered to ascertain safety of MSC application for respiratory distress.

In IPF, the first phase I trial provided data on safety of MSC application, improvements in quality of life parameters and promising progression free survival rates up to 24 months in *n* = 14 patients [112,113]. These data were confirmed in further phase I trials in *n* = 9 patients with mild or moderate IPF and *n* = 8 patients with moderately severe IPF [114,115]. The first study did not observe improvements in lung function parameters and CT scores within a follow-up period of six months [114]. However, follow-up for 48 weeks for the first time revealed hints of therapeutic improvements with slower progression of fibrosis scores measured by CT scans and slower decrease in lung diffusion capacity for carbon monoxide in those patients receiving the higher MSC dosage [116]. The second study did not detect improvements in lung function or CT fibrosis scores within six months following MSC application [115]. Stable hemodynamics after MSC application did not foster concerns from preclinical trials that MSC application to a compromised vasculature increases the risk of pulmonary embolism [114,115]. These results argue towards careful dose escalation studies in all further larger scale studies to determine the optimal dose of MSC application. Another study selected the endobronchial route of MSC administration in *n* = 14 patients. No serious adverse events including disease exacerbation were reported for the two-year follow-up period. Median progression free survival declines in lung function and deaths during follow-up were within the expected range from other epidemiologic studies [112,113]. Despite these preliminary safety results, special attention needs to be drawn to potential aggravation of lung fibrosis by the application of MSC in the chronic stage of the disease in IPF in the future.

The first study of bone marrow-derived MSC transplantation for COPD was performed in advanced stage four patients and proved the safety of MSC application. The increase in lung function 30 days after the one-time transplantation was transient and only subjective improvements in quality of life persisted [117]. The so far largest randomized phase I trial of MSC treatment for lung diseases was performed in patients with moderate and severe COPD. Overall, *n* = 62 patients were treated four times monthly and follow-up was obtained for two years. As in the former small studies, no acute toxicities or serious adverse events due to MSC infusion at a concentration of about one million cells per kilogram were recorded. Lung function testing and health questionnaire evaluation did not reveal any difference underlining the safety of the approach. Reflecting COPD disease activity, the baseline CrP values were reduced after MSC treatment. These data encourage future studies on larger cohorts of patients and re-evaluation of dosing and timing of MSC application. Although statistically not significant due to the large confidence intervals, special focus needs to be drawn to the frequency of COPD exacerbations during follow-up in subsequent trials as numeric values were higher in the intervention arm [118]. Furthermore, MSC application was evaluated in combination with standard lung volume reduction surgery and MSC were applied twice before the second step of the surgical approach without displaying any acute toxicities or serious adverse events up to 12 months of follow-up. The increase in FEV1 after 12 months was accompanied by increased expression of endothelial CD31 and anti-inflammatory IL-10 and TSG-6 in resection biopsies. Unfortunately, no control group was applied therefore a dominant beneficial effect by lung volume reduction cannot be excluded [119]. Another blinded study investigated the combined treatment with allogenic bone marrow MSC together with endobronchial valve placement. As for the other studies, no treatment related acute toxicities or adverse events were recorded within a follow-up of 90 days. While objective criteria including lung function measurements and radiologic follow-up did not demonstrate beneficial effects that can be attributed to the MSC application, again CrP values decreased and quality of life indicators improved [120]. All studies have in common that they included severely affected patients with established COPD that might have reduced the therapeutic benefit observed in the preclinical models where MSC were applied in the acute phase of the disease and during severe lung inflammation. In one subsequent phase I study with nine patients, MSC retention in the lung was prolonged in mild cases compared to severe cases. The detailed analysis of disease driving pathologies revealed tremendous changes in neutrophil and macrophage pathologies and T cell and dendritic cell subsets. No improvements in lung function were detected, but the number of hospital admissions was significantly reduced compared to the pre-intervention period [121].

For the developing lung, one first phase I study was completed so far which comprises follow-up until the age of 2 years. This study was performed in *n* = 9 extremely preterm infants requiring continuous ventilator support beyond postnatal day five for respiratory insufficiency. One of two different dosages of MSC was applied once intratracheally. This study raised great hopes as the pooled analysis of MSC application improved the pulmonary outcome using the BPD criterion at 36 weeks of gestation compared to a matched-comparison group. Of clinical relevance, pulmonary outcome until 24 months was also favorable [122,123]. The application of two slightly different dosages of MSC does not allow any conclusion about the optimal number of cells but the 10 times higher number of cells per kilogram body weight compared to the clinical studies in adults stated before might explain the positive impact on the pulmonary outcome. Going into the study details, no acute toxicities or side effects were observed. The proinflammatory cytokine response was attenuated in the tracheal aspirates following MSC administration and the respiratory severity score displayed a trend toward lower values than in control patients. Follow-up until the age of 2 years confirmed the beneficial effects of MSC application with respect to home supplemental oxygen, rehospitalizations due to pulmonary sequelae and somatic growth. Detailed analyses of psychomotor development did not reveal any disadvantage of MSC application [123].

First phase I studies were executed in other lung disease entities including bronchiolitis obliterans in *n* = 10 patients, which prevailed a striking reduction in monthly loss of forced expiratory volume to one fourth of the pre-intervention decline. Two death were recorded during the follow-up which cannot be linked to the intervention, but death rates need to be closely monitored during any future larger scale study [124]. Comprehensive summaries covering all available clinical phase I and II trials are given elsewhere [125,126,127,128].

So far, interpretation and comparison of the first completed study in men on MSC treatment are hampered by the small numbers of patients recruited, different origins of MSC, variations in MSC cell preparations and route and timing of administration, as well as inclusion criteria and study readouts [128]. The mode of MSC application is mostly intravenous, but successful endobronchial instillation was reported without detection of any safety concerns [120,122]. Significant effects on the pulmonary outcome or improvements in lung disease severity reflecting parameters were only detected after application of dosages exceeding 1 × 10^6^ cells per kilogram body weight or after repeated infusion [121,122,124]. The pending challenges and obstacles towards larger-scale MSC application within clinical trials will be discussed from a pathomechanistic and production perspective of MSC cell preparations within the next paragraph covering the safety issues and obstacles to cell-based therapies.

## 5. Safety Issues and Obstacles to Therapeutic MSC Application

It is well accepted that MSC engraftment in the lung is low. Most published studies describe disappearance within several days. Unfortunately, these studies have mostly not used state of the art molecular techniques to detect single resting cells. Persistence for several weeks has been described [85,128]. This raises concerns about the long-term safety of such an approach. Immunologic tolerance of the host to MSC poses a potential risk for malignancy; this has also not been described so far [129]. As MSC have the capability to adapt their immunologic function in an inflammatory environment with the aim to escape i.e., killing by NK cells, it remains to be determined whether this function poses a risk to immunologic cell control when MSC are applied to an inflammatory milieu [130]. Besides their mainly anti-inflammatory activity, MSC are able to trigger an initial pro-inflammatory response of the innate immune system during bacterial infection with the aim to restrict infection by attracting immune cells to the site of injury and to preserve tissue integrity [131,132,133]. MSC are able to direct the immune cells towards an optimized defense response with parallel prohibition of damage to the tissue. During bacterial infection, stimulated MSC released cytokines like IL-8 and macrophage inhibitory factor which augment antimicrobial activity while the increased release of superoxide dismutase prevents tissue damage [19]. The published literature so far on this topic does not give any hint that MSC transplanted to the lung gets mal-directed when they are exposed to the inflammatory lung milieu. This was mimicked in vitro and by the exposure to tracheal aspirates from affected patients [134]. As well, the secretion of TGF-β by MSC needs to be dissected in future studies in the context of fibrosis and attenuation of the immune system [135]. First hints from ALI rodent models raise concerns that autologous MSC transplantation from the bone marrow is associated with limited capacity of MSC for immunomodulation, resulting in reduced therapeutic efficiency compared to allogeneic transplantation [136,137].

As for any intervention therapy, MSC application poses the risk of aggravating lung injury which was recently described for the treatment of acid-induced lung injury. The authors provided an outlook on how the potential detrimental effects of aggravation of lung fibrosis by excess of TGF-β1 can be prohibited by genetic MSC modification which is addressed in detail within the next chapter [138]. Vessel occlusion in the periphery and pulmonary embolism after application are further concerns that need to be tackled within future investigations. Within the ischemia-reperfusion injury model mimicking the situation of lung transplantation, only low dose application of MSC proved beneficial while application of high doses resulted in aggregation in the microcirculation and pulmonary embolism [139]. Similar serious and lethal adverse events were recorded for high dose MSC application in other preclinical disease models leading to disseminated intravascular coagulation, respiratory and cardiovascular failure [140]. One potential solution to prevent these serious adverse events might be the anticoagulation with heparin before application of high dose MSC [140].

The augmentation of TGF-β release during disease establishment and its pro-fibrotic action is well accepted, and different experimental settings described reduction of TGF-β levels after MSC application [141]. Actually, there remain concerns that the release of TGF-β by MSC might be harmful. First functional investigations identified the modulation of immune function with an IL6 and STAT3 mediated augmentation of Treg cell proliferation and anti-fibrotic IFN-γ-inducible protein 10 as the resulting action of MSC. This is in line with the human data which did not demonstrate any harm so far [96,142,143]. To clarify the profibrotic capacity of MSC, the bleomycin IPF model is best suited as it is the most extensively studied model with respect to fibrosis induction in the lung, and timing of MSC application has been investigated in detail. In contrast to the attenuation of acute lung injury, MSC application in the chronic phase promoted lung fibrosis and the authors suggested the release of high levels of TGF-β to be responsible for aggravation of fibrotic processes [96]. Although the first study in men of MSC therapy did not show any signs of fibrosis progression, special attention needs to be drawn to this fact in all subsequent studies [115]. Similar concerns were raised in the radiation-induced lung injury model where immediate application of MSC proved beneficial, but delayed application two months after the insult promoted the development of lung fibrosis.

For the stability of MSC cell preparation, there still remain plenty of unresolved questions: MSC experience cellular and molecular changes during passaging and with increased age of the donor. This results in a decline in proliferation and functional properties including receptor expression status, proliferation, attraction to the site of injury, differentiation, release of paracrine factors and immunosuppression. These changes in MSC features were observed in preparations from different tissues including bone marrow and adipose tissue excluding a tissue-specific effect [144,145]. Although it was well documented that MSC preparations produced at one facility remain stable over time, preparations between different facilities display relevant discrepancies during cell profiling [146]. One of the indicted agents is excess reactive oxygen species production which is well known to induce a variety of cellular responses including reduced differentiation capacity, DNA damage, cellular senescence and apoptosis. The alternative use of frozen MSC cell preparations did not yield the identical therapeutic efficiency with respect to the immunomodulatory capacities after the application of freshly isolated cells. As a further obstacle, actual cell quantities easily meet the requirements for the rodent models but need to be scaled for routine use in humans [147]. To date, the technical problems are not solved to have the best and most stable products available. Research efforts urgently need to be directed towards the clinical need to have a product ready-to-use in the shelf whenever a patient is eligible for cell-based therapies [147].

## 6. Strategies to Improve Safety and Therapeutic Efficiency of MSC Based Therapies

Due to the limited efficacy of MSC application in the first clinical trials and the rare observations of side effects in preclinical studies, research directions were directed towards further improving safety and efficacy of cell-based therapies by cell transduction or cell modification before therapeutic application. During the recent years, augmentation of MSC action by genetic modification was studied in different experimental settings and for the central paracrine factors responsible for MSC action. The key findings are compiled within Table 6. ANGPT1 overexpression was one of the first to improve efficacy during ALI [64,148]. Subsequently, comparable improvements were confirmed for the growth factors KGF and HGF in mice exposed to ALI and the human lung perfusion model. Synergistic action of the two factors can be derived from the different modes of action on alveolar epithelial and endothelial cells [82,149,150]. Vice versa, gene silencing of ANGPT1, KGF or HGF significantly reduced the therapeutic efficacy of MSC therapy, confirming their central contribution to lung repair and regeneration [33,149,150,151,152]. For VEGFA, transgenic expression under an Hsp70 promoter overcame the side effects of constitutive VEGFA overexpression. MSC action was driven by Hsp70 as a marker of COPD disease severity and promoted the induction of antioxidative genes including Nrf-2, heme oxygenase-1 (HO-1), superoxide dismutase and MSC cell survival selectively at the site of injury [84]. The central role of HO-1 which is highly known for its antioxidative, anti-inflammatory and antiapoptotic effects was further secured by treatment with MSC transduced with HO-1 for ALI [153]. Its beneficial features were recapitulated, leading to increased survival and better preserved lung structure. Vice versa, gene silencing of VEGFA in ALI mitigated all features of inflammation and lung injury [154]. Similarly, FGF2 transduction augmented MSC activity. MSC transduced for the PDGF β receptor augmented growth factor effects as these MSC displayed an increased proliferation and production of ANGPT1, VEGFA, FGF and PDGF [155,156]. Overexpression of CXCR4, β-catenin or orphan receptor tyrosine kinase ROR2 enhanced chemotaxis and attraction of MSC to the site of injury in ALI and attenuated lung inflammation [157,158,159]. Transduction with the anti-inflammatory prostanoid E2 receptor or the anti-inflammatory interleukin-10 also increased the attraction and persistence of MSC at the sight of injury and augmented attenuation of lung alterations, repair and survival [63,160,161]. Overexpression of the soluble IL-1 receptor like-1, an antagonist of interleukin 33, attenuated ALI by endotoxin and promoted the direction of MSC towards an immunoregulatory phenotype with CXCR-4, TSG-6 and indoleamine 2,3-dioxygenase up-regulation which resulted in IL-10 transduction in the injured lung [62]. Knockdown of stromal cell-derived factor-1 (SDF-1) abrogated stem cell recruitment and anti-inflammatory and angiogenesis promoting capacities, assigning SDF-1 as a crucial contribution to MSC function [162]. In summary, plenty of growth promoting cytokines, anti-inflammatory factors and cell surface receptors are suitable to improve MSC recruitment to the site of injury, to stipulate the resolution of inflammation and to promote lung repair and regeneration.

Within the inflammatory response, ROS production is a central downstream action leading to tissue damage. Several approaches have been pursued to attenuate injury by oxygen radicals by transgenic overexpression of superoxide dismutase or nuclear factor like-2 (Nrf-2) which is a well-known transcription factor for antioxidant response [163,164,165]. Nrf-2 overexpression in ALI reduced epithelial cell injury, promoted MSC cell retention in the lung and promoted type II cell transdifferentiation and SPC production [165]. Claudin-4 transduction accounted for improved alveolar fluid clearance which can be further stipulated by hypoxic preconditioning of MSC [28]. Gene transduction furthermore possesses the potential to abrogate serious side effects caused by MSC application following, for example, acid induced lung injury. Transduction of MSC with IL-10 or HGF prevented lung fibrosis after MSC application as both paracrine factors are able to control the unwanted release of TGF-β1 by MSC, which accounted for the tremendous increase in lung fibrosis after naïve MSC transplantation [138].

Besides molecular modulation, MSC preconditioning is another feasible approach to improve the therapeutic efficiency of cell-based therapies and decisive preclinical studies are summarized within Table 7 [166,167,168]. As an example, pre-incubation to hypoxia or with oncostatin-M were able to augment the beneficial effects of MSC described in the bleomycin model of IPF and increased HGF secretion and survival of MSC [109,169,170]. Pharmacologic modulation is another alternative to augment MSC action. For the diabetic drug pioglitazone, pretreatment in vitro augmented the secretion of the paracrine factors VEGFA, FGF-2 and HGF in vivo [171]. Pre-incubation with n-acetylcysteine augmented the antioxidative capacity of MSC in vivo by increasing glutathione levels [172]. Pretreatment with a toll-like receptor-3 ligand inhibited microRNA [83] and enhanced MSC function by modulation of macrophage function via increased PGE2 production [173]. Similar modulation of inflammation and macrophage status was observed in the asthma model when MSC were pretreated with eicosapentaenoic acid, a polyunsaturated omega-3 fatty acid, which efficiently inhibits inflammatory responses e.g., in alveolar macrophages [174].

Another approach is the combined application of MSC together with pharmacologic therapies, which still lacks the magnitude of studies described before, but relevant findings are summarized within Table 8. Improvements in cytoprotection and preservation of lung structure were described for the combined application of MSC plus erythropoietin in BPD and the application of erythropoietin gene-modified MSC in asthma where all disease-driving pathologies were more efficiently inhibited by MSC modification [175,176]. However, combinatorial treatment also provided unexpected unfavorable results in one preclinical study of BPD where MSC application in combination with surfactant attenuated MSC cell viability and preservation of lung structure [177]. Therefore, modification of MSC cell viability and migration need to be carefully monitored during all future combinatorial treatment approaches. Nonetheless, these encouraging results from preclinical studies on MSC modification raise great hopes and will be pursued within several phase I clinical trials in the near future.

Extracellular vesicles (EV) represent the secretome of MSC containing plenty of factors and agents accounting for the therapeutic MSC efficiency. EV constitute a highly attractive alternative to direct MSC therapy as all unresolved concerns about autologous or allogenic cell transfer with respect to safety and potential malignant transformation can be left aside (summarized within Table 9). Exosomes are the most well-characterized and widely studied EVs—most of the cited studies have a size of 50–150 nm. Throughout the review, the term EV was used as general term for all types of vesicles present in the extracellular space. Features of EV action comprise all key actions of MSC function including attenuation of lung inflammation with restoration of cytokine balance, inhibition of inflammatory cell invasion and shift of immune cell phenotype towards beneficial phenotypes, as described for the M1 to M2 switch of macrophages which improves phagocytosis and simultaneously attenuates inflammation [178]. Furthermore, EV are relatively stable and protected from enzymatic degradation by their surface lipid bilayer. The therapeutic rationale is primarily based on the content of EV containing growth factors like KGF, HGF and ANGPT-1 and immunomodulating factors like TGF-β, IL-10 and PGE2. EV contain plenty of further components including gene products, transcripts and miRNAs which are dedicated to regulate and modify processes, such as cell survival and proliferation, homeostasis and differentiation, and highly impact immune functionality [178]. The latter observations are underlined by comparable therapeutic efficacy of EV or conditioned medium from MSC compared to MSC therapies in different preclinical disease models including BPD, asthma, ALI and COPD. Application of EV recapitulated the key factors and mechanistic findings identified during MSC therapy [179,180,181,182]. As described for MSC therapy before, the top candidates of paracrine action like ANGPT-1, HGF, KGF and VEGFA largely account for the therapeutic efficacy, but TSG-6 also seems to play a crucial role [181,183]. Similar results were obtained for acute lung injury, experimental asthma and COPD. Again, preserved lung histology was accompanied by better cardiovascular function parameters underlining the broad therapeutic impact on alveolar and vascular structures [33,81,184]. Presented as an illustrative example, MSC-conditioned medium proved equally efficient as MSC cell application to prevent acute lung injury by LPS in vivo and in an ex vivo human lung perfusion model underlining the central paracrine action [61,62]. However, during lung development MSC-conditioned media or EV therapy of rodents exposed to hyperoxia recapitulated all central features of MSC action including attenuation of inflammation, preservation of lung histology, lung function and cardiovascular parameters of pulmonary arterial remodeling and hypertrophy of the right ventricle [40,181,183,185]. Of note, incorporation of EV is cell type specific, at least during hyperoxic lung injury, and was detected for type II cells, lung fibroblasts and pericytes but not endothelial cells [181]. Besides the tremendous impact of immunomodulation, the interaction with resident cells of the lung comes more and more into the focus of research and was described for the inhibition of myofibroblast differentiation in IPF [186]. EV are able to stabilize important functions such as energy balance of epithelial cells and phagocytosis of bacteria by monocytes [187]. Moreover, EV possess potent antibacterial activities as previously described for MSC cell therapy [33,187]. For example, high leukotriene B4 content is a prerequisite for efficient bacterial clearance, and biochemical inhibition or knockdown of the controlling mir-145 abolished the antimicrobial activity [188].

In direct comparison between MSC and their EV treatment, disparities were also observed with respect to therapeutic efficiency. For the BPD rodent model, equivalent or even improved therapeutic efficiency of EV was described repeatedly which was traced back to the very low engraftment rates of MSC in this model [34,35,179]. EV but not their MSC were able to alleviate the increase in pulmonary small vessel medial thickness observed during BPD development [189]. In ALI comparable efficacy was detected for EV and MSC from bone marrow. In the COPD elastase model, application of human bone marrow MSC or their conditioned medium proved beneficial effects on all aspects of injury, but MSC application was superior and delayed EV application reduced the therapeutic efficiency [188,190]. In contrast, in an asthma model, EV from human adipose tissue-derived MSC showed superior activity on pro-inflammatory mediator release and inflammatory cell infiltration compared to MSC therapy on eosinophil counts, levels of TGF-β and collagen fiber content where equally modified [191]. Detailed analysis of the further available literature reveals sparse heterogeneities of MSC and EV action in a mouse model of bacterial pneumonia where the M2 macrophage shift was not detected after EV application [187]. Another example is the ex vivo human lung perfusion model mimicking the ischemia reperfusion injury during lung transplantation where lung ventilation and perfusion parameters were improved, although inflammation and growth factor action was not impacted by EV therapy [192]. The available data on general disparities in therapeutic efficiency do not retrieve an additional function of MSC cells, but differences in efficacy need to be considered with respect to the time point and effective dose of EV applied. This necessary consideration is underlined by several dose escalation studies which demonstrated enhanced therapeutic effects with increased amounts of EV [193].

As described for MSC application, EV treatment has the capacity to attenuate lung, heart and brain pathology [194]. Intravenous or intratracheal administration of EV from MSC mostly revealed comparable therapeutic efficacy that was traced back to the central role of protective factors like KGF contained in EV [33]. Therefore, gene transduction or preconditioning of MSC before harvest of EV has come into the focus of research and first studies in other disease entities besides the lung show promising results with respect to therapeutic efficacy [11]. Vice versa, inhibition of selective paracrine factors such as ANGPT-1 partly abrogated the beneficial effects of EV [195]. Production of artificial EV represents an alternative worthy vision compared to the complex production of natural exosomes. It will enable the modulation of EV function by overexpression of the FGF2 receptor which enhanced therapeutic efficiency in a preclinical model of COPD [196]. This approach might be able to overcome the therapeutic limitation to EV obtained from young donors´ MSC as EV from aged donors lack internalization into macrophages and thereby cannot execute their therapeutic efficacy as observed for ALI [197].

Different modes of exosome production, exosome dose quantification by different technical approaches and lack of standardized exosome potency assays remain unmet challenges for comparability of published studies and for stable product preparations which urgently need to be harmonized [198]. The huge amounts of EV required for therapeutic application in humans remains another unresolved challenge as the dosages required are more than factor 3000 higher than required in the rodent model [187]. Furthermore, surveillance of therapeutic safety needs to address all potential unknown long-term effects as was requested for MSC therapy previously, but overcoming these still impeding issues on the safety of cell-based therapies by the use of the secretome will pave the way to fast and broad introduction into clinical trials. The results from studies directly comparing EV and MSC therapy underline the therapeutic efficacy of EV and argue towards focusing research efforts towards optimal protocols to obtain high grade EV which can be stored frozen and can be applied as a ready-to-use product from the shelf whenever necessary.

**Table 6:** Summary of selected preclinical studies in rodents aiming to improve the therapeutic efficacy of MSC by genetic modification.

Disease Entity	Experimental Lung Disease Model	Cell Source	MSC Modification	MSC Species	Dose (Cells)	Application Route	Time Point of Application	Repeated Application	Biological Function In Vivo	Molecular Changes/Results	Reference
ALI	LPS	BM	FGF2 overexpression	mice	5 × 10^6^	i.v.	1 h p.i.	no	FGF2 overexpression better preserves lung structure and pulmonary edema	MSC overexpressing FGF2 better attenuate pro-inflammatory cytokine and MPO secretion and neutrophil infiltration	[153]
ALI	LPS	BM	CXCR4 overexpression	rat	1 × 10^6^	i.v.	1 h p.i.	no	reduced lung injury score and lung edema	enhanced mobilization and chemotaxis of MSC, increased VEGFA secretion and reduced lung inflammation	[154]
ALI	LPS	BM	β-catenin overexpression	mice	5 × 10^5^	i.t.	4 h p.i.	no	improvements in alveolar epithelial barrier integrity and lung structure impairment	better MSC retention in the lung and AEC II transdifferentiation with higher levels of KGF and IL-10 and reduced IL-1β	[155]
ALI	LPS	BM	siRNA against claudin-4	human	1 × 10^6^	i.p.	n.a.	no	Claudin-4 promotes alveolar fluid clearance	hypoxic MSC preconditioning stipulates claudin-4 secretion	[28]
ALI	LPS	BM	MSC transfected with shRNA against VEGFA	rat	5 × 10^6^	i.v.	5 h p.i.	no	attenuated anti-inflammatory properties and beneficial effects on lung injury	transfected MSC reduced the proinflammatory cytokine IL-1*β* levels and elevated the anti-inflammatory cytokine IL-10 levels	[151]
ALI	LPS	BM	shRNA HGF transfection	rat	5 × 10^6^	i.v.	5 h p.i.	no	partial abrogation of MSC effects, MSC retention in the lung was not influenced, MSC restores lung permeability and lung injury	HGF-expressing character is required for MSC to protect the injured lung	[148]
ALI	LPS	AM	Nrf2 transfected MSC	human	1 × 10^6^	i.v.	4 h p.i.	no	reduced inflammation, epithelial cell injury and fibrosis	increased cell retention in the lung, more efficient differentiation into type II cells with higher SPC content	[162]
ALI	*E. coli*	UCB	IL-10 transgentic MSC	human	1 × 10^7^/kg	i.v.	1 h p.i.	no	increased therapeutic efficiency of transgenic MSC which only prohibited all aspects of lung injury including gas exchange	enhanced macrophage function via prostaglandin E2 and lipoxygenase A4	[158]
ALI	LPS	BM	transduction with heme oxygenase-1	rat	5 × 10^5^	i.v.	2 h p.i.	no	improved survival rates, reduced lung inflammation and structural changes	superior prosurvival, antiapoptotic and paracrine functions	[150]
BPD	hyperoxia	BM	shRNA stromal cell-derived factor-1	rat	1 × 10^6^	i.t.	d7	no	reduction of beneficial MSC effects on alveolarization and angiogenesis	SDF-1 from MSC exerts anti-inflammatory and angiogenesis promoting activities	[159]
asthma	ovalbumin	BM	erythropoietin gene modified MSC	mice	n.a.	i.v.	d20	no	more efficient inhibition of all disease driving pathologies	maybe related with the downregulation of TGF-β1-TAK1-p38MAPK pathway activity	[173]
COPD	elastase	BM	VEGFA overexpression	mice	n.a.	i.v.	14 d a.t.	no	Improved attenuation of emphysema compared to naïve MSC	Increased tissue expression of VEGFA, Nrf 2 and superoxide dismutase	[84]
COPD	elastase	BM	shRNA HGF knockdown	human	0,1 vs. 5 vs. 25 vs. 125 × 10^3^/g	i.v.	6 h vs. d7 vs. d14 a.t.	no	MSC cell therapy more efficient than conditioned medium, higher doses and mid to delayed application better reduces collagen deposition and anti-inflammatory effects	anti-inflammatory, antifibrotic and antiapoptotic effects mediated partially through HGF	[187]

i.v.—intravenous; i.t.—intratracheal; i.p.—intraperitoneal; p.i.—post infection; a.t.—after treatment; MSC—mesenchymal stem cell; ALI—acute lung injury; BPD—bronchopulmonary dysplasia; COPD—chronic obstructive pulmonary disease; UCB—umbilical cord blood-derived MSC; BM—bone marrow -derived MSC; AM—amniotic-derived MSC; h – hours; d—day; n.a.—not applicable; FGF2—fibroblast growth factor 2; MPO—myeloperoxidase; VEGFA—vascular endothelial growth factor A; AEC II—alveolar epithelial cells type II; KGF—keratinocyte growth factor; IL—interleukin; HGF—hepatocyte growth factor; SDF-1—stromal cell-derived factor 1; TGF-ß—transforming growth factor beta; TAK1—transforming growth factor beta-activated kinase 1; p38MAPK—P38 mitogen-activated protein kinase; NRF 2—Nuclear factor erythroid 2-related factor 2.

**Table 7:** Summary of relevant preclinical studies in rodents intended to improve the therapeutic efficacy by MSC preconditioning.

Disease Entity	Experimental Lung Disease Model	Cell Source	MSC Modification	MSC Species	Dose (Cells)	Application Route	Time Point of Application	Repeated Application	Biological Function In Vivo	Molecular Changes/Results	Reference
ALI	endotoxin	BM	hypoxia	human	5 × 10^4^ cells/g	i.v.	n.a.	no	ischemic preconditioning potentiates the protective effect of through the secretion of exosome	less neutrophil influx and pro-inflammatory cytokine dysbalance with upregulation of IL-10	[74]
ALI	CLP	BM	preconditioning with carbon monoxide	mice	5 × 10^5^(2 h) 2.5 × 10^5^ (24 h/48 h)	i.v.	2 h, 24 h, 48 h p.i.	yes	increased survival and alleviated lung injury	preconditioning stipulates the production of proresolving lipid mediators, especially resolvins	[166]
ALI	*E. coli*	BM	MSC pretreatment with Toll-like receptor-3 agonist	human	2 × 10^7^ vs. 4 × 10^7^	i.v.	1 h p.i.	no	reduced pulmonary edema and bacterial load	increased antimicrobial activity of macrophages after application of pretreated EV	[167]
asthma	dust mite	BM	pretreatment of MSC with eicosapentaenoic acid	mice	1 × 10^5^	i.t.	1 d a.t.	no	reduced bronchoconstriction and lung tissue remodeling	reduced influx of eosinophils, macrophages, neutrophils and lymphocytes, shift towards anti-inflammatory macrophages and increased release of inflammation resolving and anti-inflammatory mediators	[171]
COPD	elastase, cigarette smoke	AT	preconditioning with pioglitazone	human	1 × 10^5^	i.v.	7 d a.t.	no	more efficient repair of lung injury	increased VEGFA production	[109]
COPD	elastase	AM	MSC predifferentiation to lung epithelial like progenitor cells	mice	1 × 10^5^	i.t.	2 weeks a.t.	no	improved lung regeneration, reduced presence of inflammatory and lung remodeling factors	integration of predifferentiated cells into lung alveolar structures	[168]
IPF	bleomycin	BM	oncostatin M preconditioning	mice	2 × 10^5^	i.t.	3 d a.t.	no	improved attenuation of inflammation, TGF-β1 and OSM induced extracellular matrix production, release of fibrotic factors	upregulation of paracrine HGF	[165]

i.v.—intravenous; i.t.—intratracheal; p.i.—post infection; a.t.—after treatment; MSC—mesenchymal stem cell; ALI—acute lung injury; COPD—chronic obstructive pulmonary disease; IPF—idiopathic pulmonary fibrosis; BM—bone marrow-derived MSC; AT—adipose tissue-derived MSC; AM—amniotic-derived MSC; CLP—cecal ligation and puncture; h—hours; d—day; EV—extracellular vesicles; n.a.—not applicable; IL—interleukin; VEGFA—vascular endothelial growth factor A; HGF—hepatocyte growth factor; TGF-ß1—transforming growth factor beta 1; OSM—oncostatin M.

**Table 8:** Summary of relevant preclinical studies in rodents intended to improve the therapeutic efficacy of MSC by co-treatment with drugs.

Disease Entity	Experimental Lung Disease Model	Cell Source	MSC Modification	MSC Species	Dose (Cells)	Application Route	Time Point of Application	Repeated Application	Biological Function In Vivo	Molecular Changes/Results	Reference
BPD	hyperoxia	BM	combined treatment with erythropoetin	mice	1 × 10^6^	i.v.	1 h before + d7	yes	improved airway structures and body weight	augmented cytoprotection	[172]
BPD	hyperoxia	PD	MSC plus surfactant	human	1 × 10^5^	i.t.	d5	no	application of surfactant partially attenuates the beneficial impact on lung histology	surfactant inhibits MSC viability and impairs the reduction of hyperoxia induced increase in mean linear intercept	[174]
asthma	ovalbumin	BM	erythropoietin gene modified MSC	mice	n.a.	i.v.	d20	no	more efficient inhibition of all disease driving pathologies	maybe related with the downregulation of TGF-β1-TAK1-p38MAPK pathway activity	[173]

i.v.—intravenous; i.t.—intratracheal; MSC—mesenchymal stem cell; BPD—bronchopulmonary dysplasia; BM—bone marrow-derived MSC; PD—placenta-derived MSC; h—hours; d—day, TGF-β1—transforming growth factor beta 1; TAK1—transforming growth factor beta-activated kinase 1; p38MAPK—P38 mitogen-activated protein kinase.

**Table 9:** Decisive preclinical studies in rodents to evaluate EV from MSC as alternative treatment option to conventional MSC application.

Disease Entity	Experimental Lung Disease Model	Source of EV	EV Modification	EV Species	EV dose (MSC Cell Equivalent)	Application Route	Time Point of EV Application	Repeated EV Application	Biological Function In Vivo	Molecular Changes/Results	Reference
ALI	*E. coli*	BM vs LF	-	human	3 × 10^6^ vs. 6 × 10^6^ vs. 9 × 10^6^	i.t. i.t. i.v.	4 h p.i.	no	increased survival, MV as effective as MSC	increased KGF release, decreased influx of inflammatory cells, pro-inflammatory cytokine release and pulmonary edema, attenuation of bacterial load	[184]
ALI	LPS (*E. coli*)	AT (young vs aged)	-	human	1 × 10^6^	i.v.	30 min p.i.	no	only EV from young donor MSC alleviated lung injury	macrophages are less prone to internalization of EV from aged MSC	[194]
ALI	*E. coli*	BM	antagomir of miR-145	human	9 × 10^6^	i.v.	4 h p.i.	no	EV and MSC decrease lung injury, efficient bacterial clearance depends on high levels of leukotriene B4	controlled by miR-145 enhanced LTB4 production and antimicrobial activity through LTB4/BLT1 signaling	[185]
BPD	hyperoxia	WJ vs BM	-	human	0.5 × 10^6^	i.v.	d4	no	reduction of alveolar and vascular remodeling and lung fibrosis same for WJ and BM EV	shift from pro-inflammatory M1 macrophage status to anti-inflammatory M2 status	[176]
BPD	hyperoxia	UCB	siRNA VEGF-knockdown	human	5 × 10^5^	i.t.	d14	no	EV similar effective in reduction of impairments in alveolarization and vasculogenesis	knockdown of VEGFA abolished the beneficial effects, EV were internalized into type II cells, fibroblasts and pericytes, but into endothelial cells	[178]
BPD	hyperoxia	UCB + WJ	TSG-6 siRNA knockdown	human	0.7x10^6^	i.p.	d2 + d4	yes	EV similar effectiveness in reduction of lung, heart and brain pathology	knockdown of TSG-6 abrogated the beneficial effects	[180]
BPD	hyperoxia	UCB	-	human	6 × 10^6^	i.t.	d3, d7, d10	yes	only EV, but not MSC prevented an increase in medial thickness in small pulmonary arteries	further studies required for mechanism of action	[186]
asthma	ovalbumin	AT	-	human	1 × 10^5^	i.v.	d47 (1d after last challenge)	no	superiority of EV compared to MSC with respect to pro-inflammatory mediators and inflammatory cell infiltration, but similarly reduced eosinophils, collagen fiber content and levels of TGF-β	further studies required for mechanism of action	[188]

i.v.—intravenous; i.t.—intratracheal; i.p.—intraperitoneal; p.i.—post infection; MSC—mesenchymal stem cell; ALI—acute lung injury; BPD—bronchopulmonary dysplasia; UCB—umbilical cord blood-derived MSC; BM—bone marrow-derived MSC; AT—adipose tissue- derived MSC; AM—amniotic-derived MSC; WJ—whartons’s jelly-derived MSC; LF—lung fibroblasts; h—hours; min—minutes; d—day; EV—extracellular vesicles; miR-145—microRNA 145; LTB4—leukotriene B4; KGF—keratinocyte growth factor; VEGFA—vascular endothelial growth factor A; TGF-ß—transforming growth factor beta.

## 7. Conclusions and Perspectives

Pulmonary diseases across ages pose an ever-increasing therapeutic challenge to clinicians´ therapy. Research efforts during the last decade provided tremendous progress towards the pathophysiologic understanding of disease origins and plenty of targeted therapies were evaluated within preclinical and clinical settings [199,200]. Despite a number of common features, different disease entities and disparate disease subtypes require different targeted approaches to specifically tackle the central disease drivers. This magnitude of different approaches poses a challenge to researchers and industrial partners with respect to feasibility and costs. Furthermore, lung diseases with lower incidences or reduced acute disease burdens might fall out of the research focus. Therefore, broadly applicable therapies remain the ultimate vision. During the recent years, two central disease pathologies have been unraveled which have a common origin: lung stem cell phenotype distortion, and stem cell rarefication and pathologic changes in the local microbiome. While for the latter interventional approaches are already introduced successfully into the clinics, MSC-based therapies are still mainly studied in the laboratory [4,5,193,200,201]. The use of allogenic MSC seems fascinating and the here summarized preclinical results raise great hopes that MSC-based therapies will successfully lead to cures rather than alleviation of disease symptoms. The available data from clinical trials proved the safety of such an approach across ages and disease entities. They deliver the groundwork to initiate larger scaled trials with the aim to prove not only feasibility but efficacy. As for any new therapy, the beginnings of MSC therapy were slow and difficult but now a crucial moment has been reached where focused research efforts will pave the way to success in the near future. From our perspective, a ten-point plan needs to be addressed to enable the broad clinical introduction of MSC-based therapies to all forms of lung diseases:

1. Global registries and databanks meeting data protection requirements need to be established where research results can be shared immediately between researchers. Scientific publishers need to find solutions to honor this willingness to speed up the scientific progress.

2. Effects and side effects need to be studied using harmonized protocols reflecting the clinical situation. It needs to be taken into account that cell-based therapies will initially be reserved to the most severely affected patients and chronic courses where inflammation might no longer be the leading driver of disease progression or where structural pathologies have been already established. If preclinical data underline the need for early disease interventions, the best candidates for a cell-based approach need to be identified by clinical measures and biomarkers.

3. Studies need to be extended to different animal models and animal strains within one species to account for the inter-individual genetic variability and to different species including primates that best reflect the structural characteristics and developmental stages in humans.

4. The search for the optimum MSC cell origin needs to address the source and age of MSC. Although MSC from the umbilical cord blood and Wharton´s Jelly are available on a large scale, the growing number of patients and indications as well as the reluctance of parents to provide their infant´s regenerative toolkits to the general public will sooner or later lead to a shortage of cells.

5. EV overcome the obstacles of allogenic cell-based therapies and therefore research efforts should be directed towards the optimum EV product and sufficiently scaled production capacities. These efforts need to include the identification of suitable assays to determine product quality and stability.

6. Artificial production of EV needs to be tackled as large-scale production of EV has the potential to overcome natural product variations and to provide sufficient amounts of EV to bypass the concerns of stable therapeutic efficacy.

7. Therapies usually display improved therapeutic efficacy in the artificial laboratory setting than in the complex clinical situation of patients. Therefore, efforts specifically designed to improve the therapeutic efficacy and to abrogate potential side effects are of great therapeutic potential as described for genetic transduction and preconditioning of MSC.

8. New at best in vitro and high throughput assays need to be developed that cover all aspects of MSC action to enable the timely and comprehensive analysis of all classical actions including paracrine factors and the transfer of cellular components.

9. The short-term recruitment of MSC to the lung argues towards repeated applications. As for many therapies, a one-time application does not lead to therapy success. Cell-based therapies need to be investigated for repetitive or even permanent applications in chronic lung diseases with the aim to stabilize lung function and to alleviate disease symptoms.

10. Gender specific effects have been described throughout all disease entities and across ages. They need to be addressed with respect to cell product preparation and gender-dependent therapeutic efficacy in patients treated with cell-based therapies.

## Figures and Tables

**Table 1:** Summary of important preclinical studies in rodents examining the efficacy of MSC to prevent/treat bronchopulmonary dysplasia.

Experimental Lung Disease Model	Species	Cell Source	MSC Species	Dose (Cells)	Application Route	Time Point of Application	Repeated Application	Biological Function In Vivo	Molecular Changes/Results	Reference
hyperoxia	rat	UCB vs. AT vs. MNC	human	5 × 10^5^	i.t.	d5	no	UC MSC better preserve lung structure than AT or MNC MSC	higher HGF and VEGFA production in BM MSC, only BM MSC attenuate impaired angiogenesis, cell death induction, macrophage influx and pro-inflammatory cytokine production	[42]
hyperoxia	rat	UCB	human	5 × 10^5^ 2 × 10^6^	i.t. i.v.	d5	no	reduction of lung inflammation	intratracheal application more efficient than intravenous injection	[43]
hyperoxia	rat	BM	rat	1 × 10^6^	i.t.	d7	no	improved alveolarization and vascular density, reduced pulmonary hypertension	MSC from female donors stronger impact on vascular development, maybe the most potent MSC population for lung repair in severe BPD	[37]
hyperoxia	mice	BM	human	2.5 × 10^5^	i.t.	d4	no	attenuated structural damage and lung fibrosis until adulthood	shift in macrophage populations towards an anti-inflammatory phenotype	[38]
hyperoxia	mice	BM	mice	5 × 10^4^	i.v.	d4	no	cytoprotective effects and attenuation of lung injury	paracrine MSC reaction via the release of immunomodulatory factors to ameliorate the parenchymal and vascular injury	[34]
hyperoxia	rat	BM	rat	1 × 10^5^	i.t.	d4	no	improved survival and exercise tolerance while attenuating alveolar and lung vascular injury and pulmonary hypertension	BM MSC prevent arrested alveolar and vascular growth in part through paracrine activity	[35]

i.v.—intravenous; i.t.—intratracheal; MSC—mesenchymal stem cell; BPD—bronchopulmonary dysplasia; UCB—umbilical cord blood-derived MSC; BM—bone marrow-derived MSC; AT—adipose tissue-derived MSC; MNC—umbilical cord blood mononuclear cells; d—day; HGF—hepatocyte growth factor; VEGFA—vascular endothelial growth factor A.

**Table 2:** Summary of decisive preclinical studies in rodents examining the therapeutic potential of MSC to treat asthma.

Experimental lung Disease model	Species	Cell Source	MSC Species	Dose (Cells)	Application Route	Time Point of Application	Repeated Application	Biological Function In Vivo	Molecular Changes/Results	Reference
house dust mite	mice	BM	mice	1 × 10^6^	i.v.	d14	no	reduced airway hyperresponsiveness	reduction of eosinophilia, Th2 response and activated dendritic cells	[48]
ovalbumin	mice	BM	mice	1 × 10^6^	i.v.	d19	no	reduced pulmonary inflammation	reduced attraction of lymphocytes and eosinophils to the lung, suppression of lung dendritic cell maturation and Th2 response	[49]
ovalbumin	mice	BM	mice	1 × 10^6^	i.v.	d67	no	combination therapy of MSC plus simvastatin potentiates anti-inflammatory effects and suppression of lung airway remodeling	reduced recruitment of neutrophils and eosinophils, goblet cell hyperplasia and lung fibrosis	[50]
house dust mite	mice	BM	mice	5 × 10^5^	i.v.	d27	no	decreased airway responsiveness, reduced eosinophil and neutrophil influx, normalized lung function	MSC express high levels of COX, M2 macrophages high levels of IL-10 and TGF-β and low level of IL-6	[55]
ovalbumin	mice	UCB	human	5 × 10^5^	i.v.	d14	no	decreased airway hyperresponsiveness, inflammatory cell infiltration and Th2 cytokine production while percentage of Tregs was increased	MSC treatment reduces allergic inflammation, which could be mediated by regulatory T cells	[51]
ovalbumin	mice	BM vs. AT	mice	1 × 10^5^	i.t.	d22	no	reduction of lung parenchymal inflammation and inflammatory profile of alveolar macrophages	therapeutic efficiency only after treatment with BM MSC	[54]
ovalbumin	rat	PD	human	1 × 10^6^/kg	i.v.	d7	no	inflammatory cell infiltration and goblet cell hyperplasia were markedly decreased	shift from Notch-1, -2 and jagged-1 to Notch-3, -4 and delta-like ligand-4 signaling	[57]
ovalbumin	mice	iPSC vs. BM	human	1 × 10^6^	i.v.	d0 (iMR90-iPSC) + d20 (all)	yes	inhibition of inflammatory cell infiltration and mucus production, reduction in eosinophil infiltration, and a decrease in inflammatory cell infiltration	iPSC-MSC same therapeutic effect as BM MSC	[44]
ragweed	mice	BM	mice	n.a.	i.v.	d14	no	inhibition of eosinophil infiltration, decreased levels of Th2 cytokines and immunoglobulins, IL-4 and/or IL-13 activate the STAT6 pathway in the BMSCs resulting in an increase of their TGF-β production	BM MSC suppress Th2-driven allergic responses by TFG- β production	[45]
house dust mite	mice	BM	mice	1 × 10^5^	i.t.	d22	no	reducing levels of IL-4, IL-13, and eotaxin, increased mRNA expressions of TGF-β1, IFN-γ, IL-10, TSG-6, IDO-1, and IL-1RN and induced M2 macrophage polarization	reduction of inflammation and remodeling, as well as improvement in lung function	[58]

i.v.—intravenous; i.t.—intratracheal; MSC—mesenchymal stromal cell; UCB—umbilical cord blood-derived MSC; BM—bone marrow-derived MSC; AT—adipose tissue-derived MSC; PD—placenta-derived MSC; iPSC—induced pluripotent stem cells; d—day; IL—interleukin; COX—cyclooxygenase; TGF-ß—transforming growth factor beta; IFN-y—interferon gamma; TSG-6—tumor necrosis factor-inducible gene 6 protein; IDO-1—indolamin-2,3-dioxygenase; Th2—t helper cell type 2.

**Table 3:** Summary of key preclinical studies in rodents on the treatment of acute lung injury with MSC.

Experimental lung Disease Model	Species	Cell Source	MSC Species	Dose (Cells)	Application Route	Time Point of Application	Repeated Application	Biological Function In Vivo	Molecular Changes/Results	Reference
LPS	rat	BM	rat	5 × 10^5^	i.v.	4 h p.i.	no	reduction of lung edema	RNA interference against KGF abrogated the MSC effect	[65]
Klebsiella	mice	(PαS) BM	mice	1 × 10^6^	i.v.	4 h p.i.	no	reduced alveolitis and lung edema	attenuated neutrophil, T cell and dendritic cell influx	[77]
Pseudomonas aeruginosa	mice	AT	mice	1 × 10^5^ vs. 1 × 10^6^	i.t.	1 h p.i.	no	reduced inflammation, neutrophil accumulation, bacterial burden and lung injury	protective effects only be achieved at high dose instillation, inhibition of prostaglandin E2 production by IGF-1, improved bacterial properties and phagocytosis activity in macrophages	[23]
*E. coli*	rat	BM	human	1 × 10^7^ vs. 2 × 10^7^ vs. 5 × 10^6^ vs. 2 × 10^6^/kg	i.v. vs. i.t.	30 min p.i.	no	intratracheal application as effective as intravenous application, reduced efficiency of cryopreserved cells, best balance between efficacy and dose was seen at 1 × 10^7^ hMSCs/kg	increased macrophage phagocytosis capacity and LL-37 secretion	[67]
Influenza	mice	BM	human	5 × 10^5^	i.v.	5 d p.i.	no	reduced impairment of alveolar fluid clearance and attenuated lung injury	effects were mediated by infected cells’ release of soluble factors that down-regulate the sodium and chloride transporters	[73]
Influenza	mice	BM	mice	1 × 10^5^	i.v.	30 min p.i.	no	reduced lung injury, pro-inflammatory cytokine production, inflammatory cell recruitment and lung edema	reduction of JNK and ERK phosphorylation	[74]
*E. coli*	mice	BM	human	1 × 10^6^	i.v. vs. i.n.	4 h p.i.	no	beneficial effects centrally exerted by enhanced alveolar macrophage phagocytosis which is stipulated by mitochondria transfer via nanotube structures	macrophage depletion abolished the beneficial MSC effects, i.v. route could be more beneficial	[68]
*E. coli*	rat	BM	human	1 × 10^7^/kg	i.v.	30 min p.i.	no	reduction in bacterial load and lung inflammation, attenuated lung injury, potential to enhance epithelial wound repair	CD362+ MSC account for the therapeutic efficiency	[70]
influenza	mice	UCB vs. BM	human	5 × 10^5^	i.v.	5 d p.i.	no	improved outcome for umbilical cord MSC compared to BM MSC	umbilical cord MSC improved growth factor release of ANGPT-1 and HGF	[75]
*E. coli*	mice	BM	mice	75 × 10^4^	i.t.	4 h p.i.	no	down-regulation of proinflammatory responses, reducing TNF-α and MIP-2 and increasing the anti-inflammatory cytokine IL-10	BM MSC decrease the severity of endotoxin-induced ALI and improve survival	[60]

i.v.—intravenous; i.t.—intratracheal; i.n.—intranasal; p.i.—post infection; MSC—mesenchymal stem cell; ALI—acute lung injury; UCB—umbilical cord blood-derived MSC; BM—bone marrow -derived MSC; (PαS) BM—double positive PDFGRα+ SCA1+ bone marrow-derived MSC; h—hours; min—minutes; d—day; KGF—keratinocyte growth factor; IGF—Insulin-like growth factor; ANGPT-1—angiopoietin 1; TNF-α—tumor necrosis factor alpha; MIP-2—macrophage inflammatory protein-2; IL—interleukin; JNK—c-JUN-N-terminal kinase; ERK—extracellular signal-regulated kinase.
